# Intractable Vomiting Secondary to Gastric Outlet Obstruction by a Duplication Cyst

**DOI:** 10.1097/PG9.0000000000000187

**Published:** 2022-03-17

**Authors:** Javed Faiza, Teresa Rivera-Penera, Nishith Bhattacharyya

**Affiliations:** From the * Department of Pediatric Gastroenterology and Nutrition, St. Joseph’s Children’s Hospital, St. Joseph Regional Medical Center, Paterson, NJ; † Division of Pediatric Gastroenterology and Nutrition, St. Joseph’s Children’s Hospital, Paterson, NJ; ‡ Division of Pediatric Surgery, St. Joseph’s Children’s Hospital, Maplewood, NJ.

**Keywords:** intractable vomiting, gastric outlet obstruction, gastric duplication cyst

## Abstract

Gastric duplication cysts are a rare finding in the adult population. Duplication cysts comprise 4% of the alimentary tract duplications, and about 67% are usually discovered within the first year of life. They can be located anywhere in the gastrointestinal tract, with the majority located in the greater curvature of the stomach. Duplication cysts may be identified on imaging studies in asymptomatic patients or may present with nonspecific symptoms that can include emesis and abdominal pain. We describe a case of a communicating cyst in the antrum of the stomach leading to gastric outlet obstruction in a 19-year-old male.

## INTRODUCTION

Duplication of the alimentary tract is a relatively rare congenital anomaly. It can affect any part of the gastrointestinal tract with ileum being the most common site. Gastric duplication cysts comprise 4% of all gastrointestinal duplications. We present a case of a communicating duplication cyst in the gastric antrum leading to gastric outlet obstruction in an older teenage boy.

## CASE PRESENTATION

A 19-year-old male with a medical history of mild intermittent asthma presented in the emergency department with epigastric pain associated with vomiting for 2 weeks. Symptoms worsened and the patient was unable to tolerate anything orally, having lost 3 pounds. Blood tests for complete blood count and chemistries were unremarkable. Ultrasound of the abdomen showed a cystic structure adjacent to the gastric pylorus. The patient underwent computed tomographic (CT) scan of the abdomen and pelvis that revealed a 6.4 × 3.2-cm cystic structure in the distal gastric antrum adjacent to the gastric pylorus, concerning for partial gastric obstruction.

**FIGURE 1.**
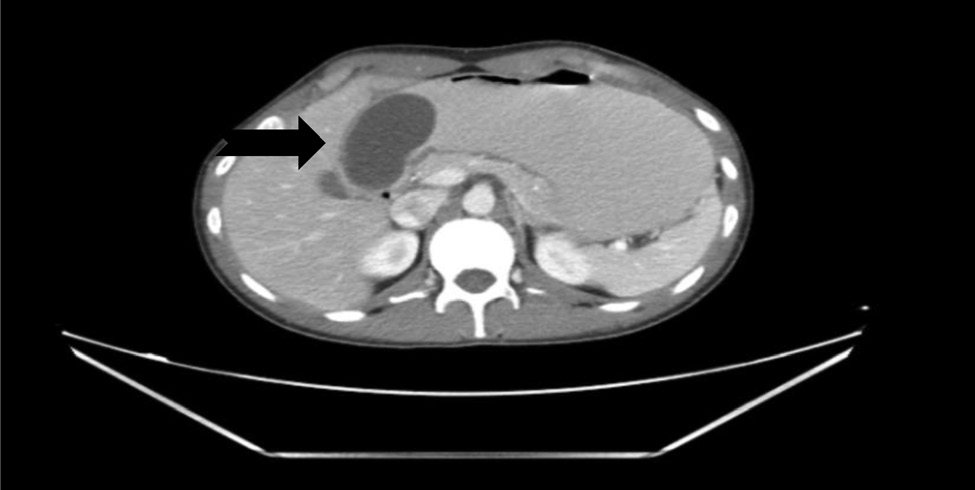
Computed tomographic scan showing 6.4 × 3.2 cm cystic structure in the distal stomach antrum adjacent to the pylorus producing partial gastric outlet obstruction.

Adult Surgery and Pediatric Gastroenterology were consulted, and he was admitted to the pediatric floor.

Esophagogastroduodenoscopy revealed an extrinsic mass pressing against the gastric antrum. A liter of fluid was aspirated from stomach. Linear erosions were found, consistent with prolapse gastropathy from vomiting.

Exploratory laparotomy revealed a large cyst (5.1 × 1.2 × 1.2 cm) obstructing the pylorus. The cyst was excised completely, leaving behind the posterior wall that was communicating with the duodenum and pylorus.

Histology revealed antral-type gastric mucosa with focally prominent chronic, rudimentary layer of muscularis propria and outer surface with fibroblastic proliferation and amorphous material deposition of fibrin, findings consistent with gastric duplication cyst. The patient’s postoperative course was uneventful.

**FIGURE 2.**
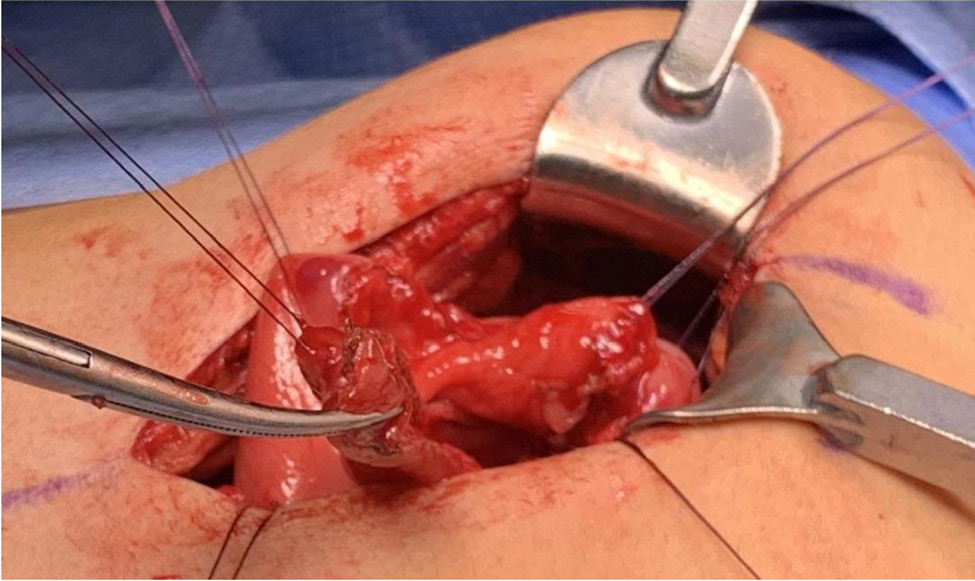
Exploratory laparotomy showed a large cystic mass obstructing the pylorus. It was resected and sent for pathology.

## DISCUSSION

Gastrointestinal duplication is a relatively rare anomaly that may occur throughout the gastrointestinal tract, from the oral cavity to the rectum, with ileum being the most common site. Duplications of the digestive tract can be observed with an incidence of 1:4500 for the general population. They can be found along the entire scope of the digestive tract. Gastric duplications typically become symptomatic during childhood; 67% are diagnosed within the first year of life, and less than 25% are discovered after 12 years of age. Gastric duplication cysts are a rare phenomenon and account for only 2% to 9% of all gastrointestinal duplications.^1,3^

Approximately 80% of gastric duplications are cystic and do not connect with the stomach lumen. The rest are tubular with some communication. When the lumens are contiguous, the structure is described as tubular, and when the lumens are not contiguous with the stomach lumen, it is described as cystic. The mucosal lining of duplication may be histologically comparable to the gut segment to which it is topographically connected. Some duplications, on the other hand, may comprise lining from other parts of the digestive or respiratory system. The presence of respiratory epithelium in cysts of the thorax, tongue, liver, and stomach implies that the foregut’s undifferentiated epithelium might undergo transition to differentiated specialized epithelium during the embryonic period.^4,5^

The clinical presentation of gastric duplication cysts can be highly variable and nonspecific ranging from vague abdominal pain to nausea, vomiting, epigastric fullness, weight loss, anemia, dysphagia, dyspepsia with abdominal tenderness, and epigastric mass on physical examination. The cysts may also be manifested by complications such as infection, gastrointestinal bleeding, perforation, ulceration, fistula formation, obstruction, compression, pancreatitis, or carcinoma arising in the cysts.^6–8^

In our patient, the duplication cyst was communicating, which could explain the lack of symptoms for many years before presentation. Blockage of the communication, possibly from anatomical shifting, may have caused the cyst duplication to become significantly enlarged to cause the gastric outlet obstruction.

Although it is difficult to diagnose gastric duplication cysts preoperatively, recent imaging modalities have provided some informative findings. Computed tomography scan and endoscopic ultrasound are the best ways to identify gastric duplication cysts. Contrast-enhanced CT scan typically demonstrates gastric duplication cysts as a thick-walled cystic lesion with enhancement of the inner lining. Calcification is occasionally observed on CT. These findings are of diagnostic significance for gastric duplication cysts.^1,2,6^

Complete removal of the duplication is the preferred treatment to avoid the risk of possible complications such as obstruction, torsion, perforation, hemorrhage, and malignancy. A noncommunicating gastric duplication cyst is classically treated by complete excision of the cyst and resection of the shared wall between stomach and the duplication cyst. Communicating gastric duplication cysts usually require no intervention when both gastric lumens are patent. Drainage and marsupialization of the cyst have been suggested.^1,6^

Although rarely seen in the young adult population, congenital gastric cysts should be considered in the differential diagnosis of patients with intractable vomiting and symptoms of gastric outlet obstruction for prompt diagnosis and management.

## References

[1] Singh JP Rajdeo H Bhuta K Savino JA. Gastric duplication cyst: two reports and review of the literature: Hindawi Publishing Corporation-Case reports in Surgery 2013, Article ID 605059. View at: Publisher Site | Google Scholar 10.1155/2013/605059 PMC3590563 23509656

[2] Kuraoka K Nakayama H Kagawa T Ichikawa T Yasui W. Adenocarcinoma arising from a gastric duplication cyst with invasion to the stomach: a case report with literature review. J Clin Pathol. 2004;57:428–431. 15047751 10.1136/jcp.2003.013946 PMC1770274

[3] Doepker MP Ahmad SA. Gastric duplication cyst: a rare entity. J Surg Case Rep. 2016;2016:rjw073. 27150283 10.1093/jscr/rjw073 PMC4858351

[4] Kim DH Kim JS Nam ES Shin HS. Foregut duplication cyst of the stomach. Pathol Int. 2000;50:142–145. 10792773 10.1046/j.1440-1827.2000.01008.x

[5] Murakami S Isozaki H Shou T Sakai K Toyota H. Foregut duplication cyst of the stomach with pseudostratified columnar ciliated epithelium. Pathol Int. 2008;58:187–190. 18251783 10.1111/j.1440-1827.2007.02209.x

[6] D’Journo XB Moutardier V Turrini O. Gastric duplication in an adult mimicking mucinous cystadenoma of the pancreas. J Clin Pathol. 2004;57:1215–1218. 15509688 10.1136/jcp.2004.019091 PMC1770488

[7] Lavine JE Harrison M Heyman MB. Gastrointestinal duplications causing relapsing pancreatitis in children. Gastroenterology. 1989;97:1556–1558. 2684724 10.1016/0016-5085(89)90404-6

[8] Gugig R Ostroff J Chen YY. Gastric cystic duplication: a rare cause of recurrent pancreatitis in children. Gastrointest Endosc. 2004;59:592–594. 15044909 10.1016/s0016-5107(04)00011-2

